# Impact of the COVID-19 Pandemic on General Hospital Physicians Work and Mental Health: An International Cross Sectional Study

**DOI:** 10.1192/j.eurpsy.2022.149

**Published:** 2022-09-01

**Authors:** D. Telles Correia, F. Novais, G. Mattei

**Affiliations:** 1 FACULDADE DE MEDICINA UNIVERSIDADE DE LISBOA, Psychiatry, Lisboa, Portugal; 2 Lisbon University, Faculty Of Medicine, Lisbon, Portugal; 3 Modena University, Psychiatry, Modena, Italy

**Keywords:** COVID-19, Psychiatrists, General Physician, Mental Health

## Abstract

COVID-19 pandemic had an important impact in mental health across all countries and populations. However, health care professionals, particularly those in the front line have been subjected to increased levels of stress, workload, deterioration of work environment and working conditions while potentially being afraid of contracting the infection themselves or infecting love ones due to the higher risk of contagion when dealing with infected patients. Some studies have stressed out this impact showing increased levels of burnout, depression, hopelessness, stress and post-traumatic stress in all physicians however, the impact of the pandemic may have been different depending on the specialty. We intended to study the impact of COVID-19 pandemic for doctors working at general hospitals and liaison psychiatrists dealing with COVID-19 patients in Europe. We developed and applied online questionnaires to physicians working at general hospitals and psychiatrists working at liaison services, in different European countries (Portugal, Italy, Belgium, Greece, Poland, Croatia), in order to determine what were their working conditions and it they reported mental health symptoms during the pandemic. This questionnaire included demographic data, questions about working conditions when dealing with general and COVID patients and the Hospital Anxiety and Depression scale (HADS). It was distributed thought email and social media platforms used by doctors. This work has been approved by each local Ethics committee and all participants signed an informed consent.

**Disclosure:**

No significant relationships.

